# Archives of Iranian Medicine Journal (2010–2024)

**DOI:** 10.34172/aim.31751

**Published:** 2024-09-01

**Authors:** Mohammad Hossein Azizi, Shokoofeh Borzabadi

**Affiliations:** ^1^Academy of Medical Sciences IR, Iran, Tehran, Iran

 The *Archives of Iranian Medicine Journal* (AIM) was launched in October 1998. Totally, 205 issues have been published from the beginning until the end of 2023. Now, AIM is around 26 years old and indexed in several international and local databases including: MEDLINE, EMBASE, PASCAL, CSA, SID (since 2006) and Web of Science (since 2007). In the last fourteen years, the editorial office of the AIM journal received 16 216 manuscripts from Iran and abroad and over 25.7% of articles were from Turkey, China, India, Pakistan, Tunisia, Saudi Arabia, Serbia, Algeria, Yemen, Iraq, UAE, Ukraine and other countries. The average acceptance rate was 15.8%. After the initial evaluation, all received papers are peer reviewed by at least two experts as well as a biostatistics specialists. They are also checked for scientific misconducts according to the Committee of Publication Ethics (COPE) policy. The AIM journal is a COPE member since 2011. During the period from 2010 to 2024, we have had 675 weekly editorial meetings, and 948 peer reviewers in Iran or abroad who evaluated the manuscripts which were selected by the editorial team of the AIM Journal.

 In addition, the AIM journal has published more than 10 special issues on hot topics from various scientific and research centers in Iran including:

- The National and Sub-National Burden of Disease (NASBOD), 1990 – 2013, Volume 17, Number 3, March 2014
- Cancer in Iran. Volume 17, Number 4, April 2014
- Global Burden of Disease (GBD) Study in Iran, 2010. Volume 17, Number 5, May 2014
- Non-Communicable Disease. Volume 17, Number 6, June 2014
- National and Subnational Burden of Chronic Disease in Iran. Volume 17, Number 12, December 2014
- Burden of Disease in Iran: Perspective of the GBD Study 2010.Volume 18, Number 8, August 2015
- Book of Abstracts The 3rd International Congress of Transfusion Medicine, Evidence-Based Use of Blood Components and Plasma Derived Medicines, Dec 15-17, 2015, Tehran, Iran. Volume 18, Number 12 (suppl 1), December 2015
- Mental Health. Volume 20, (11 Suppl. 1), November 2017
- Peace through Health. Volume 23, No.4 (Special Issue), April 2020
- COVID-19 Special Issue. Volume 23, (COVID-19 Special Issue), April 2020

Additionally, the AIM journal has so far published four books on the history of medicine in Iran, consisting of collections of papers previously published in AIM.^1-4^

 After the emergence of the COVID-19 pandemic, from September 2022 until March 2024, we received 1454 manuscripts on COVID-19, of which 93 papers appeared in regular monthly issues of the AIM and in 2020, an additional special issue on COVID-19 was also published. These papers have been cited 1542 times, as reported by Scopus and 832 times according to PubMed Central (PMC).

 The impact factor (IF) of the AIM was 3.138 in 2022, but in 2023, it was decreased to 2.1 during the COVID-19 pandemic which had negative impacts on the scientific activities of the journals worldwide,^5^ despite the fact that health journals published many papers on COVID-19 which temporarily increased their IF.

 The journal H- index was 61 between 2022 and 2023. In 2022, the Q index (Journal Citation Ranking and Quartile Score) was Q2, but again changed to Q3 in 2024. According to the AIM Journal site, the abstracts of the published papers were viewed 4 359 669 times between 2010 and 2024.^6^

 Last but not least, the editorial team tries to promote the journal scientific quality by publishing the best received papers and welcomes the authors to submit their articles. Furthermore, the respected readers can help us by providing their feedback with regard to the quality of the published papers (E-mail address of the Journal: AIM@journalaim.com)
